# Maternal glucocorticoids do not directly mediate the effects of maternal social stress on the fetus

**DOI:** 10.1530/JOE-22-0226

**Published:** 2022-10-18

**Authors:** Ying Sze, Joana Fernandes, Zofia M Kołodziejczyk, Paula J Brunton

**Affiliations:** 1 Centre for Discovery Brain Sciences, University of Edinburgh, Edinburgh, UK; 2 The Roslin Institute, University of Edinburgh, Easter Bush Campus, Midlothian, UK; 3 Zhejiang University-University of Edinburgh Institute, International Campus, Haining, Zhejiang, P.R. China

**Keywords:** prenatal stress, placenta, pregnancy, sex differences, glucocorticoids

## Abstract

Stress during pregnancy negatively affects the fetus and increases the risk for affective disorders in adulthood. Excess maternal glucocorticoids are thought to mediate fetal programming; however, whether they exert their effects directly or indirectly remains unclear. During pregnancy, protective mechanisms including maternal hypothalamic–pituitary–adrenal (HPA) axis hyporesponsiveness and placental 11β-hydroxysteroid dehydrogenase (11βHSD) type 2, which inactivates glucocorticoids, limit mother-to-fetus glucocorticoid transfer. However, whether repeated stress negatively impacts these mechanisms is not known. Pregnant rats were exposed to repeated social stress on gestational days (GD) 16–20 and several aspects of HPA axis and glucocorticoid regulation, including concentrations of glucocorticoids, gene expression for their receptors (*Nr3c1*, *Nr3c2*), receptor chaperones (*Fkbp51*, *Fkbp52*) and enzymes that control local glucocorticoid availability (*Hsd11b1*, *Hsd11b2*), were investigated in the maternal, placental and fetal compartments on GD20. The maternal HPA axis was activated following stress, though the primary driver was vasopressin, rather than corticotropin-releasing hormone. Despite the stress-induced increase in circulating corticosterone in the dams, only a modest increase was detected in the circulation of female fetuses, with no change in the fetal brain of either sex. Moreover, there was no change in the expression of genes that mediate glucocorticoid actions or modulate local concentrations in the fetal brain. In the placenta labyrinth zone, stress increased *Hsd11b2* expression only in males and *Fkbp51* expression only in females. Our results indicate that any role glucocorticoids play in fetal programming is likely indirect, perhaps through sex-dependent alterations in placental gene expression, rather than exerting effects via direct crossover into the fetal brain.

## Introduction

Stress experienced during pregnancy has detrimental effects on the offspring across the life course, beginning during fetal development, persisting through the postnatal period and into adulthood (Glover *et al.* 2018). This phenomenon is known as fetal programming, where changes during fetal development bring about long-lasting effects (Barker 1990). In women, maternal anxiety or distress during pregnancy is associated with a greater risk of psychiatric or affective disorders in the offspring (Entringer *et al.* 2015, O'Donnell & Meaney 2017). Wide-ranging effects of prenatal stress are also reported in animal studies, with adult offspring displaying phenotypes including increased anxiety- and depressive-like behaviour, as well as cognitive and social deficits (Brunton 2013, Maccari *et al.* 2014).

Concomitant with these affective disorders and aberrant behavioural phenotypes, dysregulation of the hypothalamic–pituitary–adrenal (HPA) axis is frequently observed (Pariante & Lightman 2008). The HPA axis is the primary neuroendocrine stress response system. It is activated following perturbations to normal homeostasis, resulting in increased secretion of glucocorticoids from the adrenal gland, which mobilises energy stores to effectively deal with the stressor. Given glucocorticoids have wide-ranging effects on gene expression, metabolism and neural function, excess or inappropriate glucocorticoid action can be detrimental (Sapolsky *et al.* 2000). Therefore, HPA axis activity is tightly regulated by a glucocorticoid negative feedback mechanism at the level of the anterior pituitary gland, hypothalamus and hippocampus, mediated via glucocorticoid receptors (NR3C1) and mineralocorticoid receptors (NR3C2) (Ulrich-Lai & Herman 2009). Local glucocorticoid availability in target organs is further controlled through the actions of 11β-hydroxysteroid dehydrogenase (HSD11B) enzymes, which interconvert glucocorticoids between their active and inactive 11-ketosteroid forms. Glucocorticoid–NR3C1 interaction is also modulated by the chaperone proteins, FK506-binding protein 51 (FKBP51) and 52 (FKBP52), which regulate receptor-ligand binding and nuclear translocation of NR3C1 (Wochnik *et al.* 2005). FKBP51 is a negative regulator of NR3C1 function, reducing the binding affinity of NR3C1 for glucocorticoids and sequestering NR3C1 in the cytoplasm; whereas FKBP52 positively modulates NR3C1 function, increasing the binding affinity of NR3C1 for glucocorticoids and facilitating NR3C1 nuclear translocation (Baker *et al.* 2018). Hence, changes affecting these mechanisms that mediate glucocorticoid negative feedback, regulate glucocorticoid availability and modulate NR3C1 function could contribute to the HPA axis dysregulation observed following prenatal stress.

In our model of repeated maternal social stress, the prenatally stressed adult offspring display heightened anxiety-like behaviour and exaggerated HPA axis responses to both physical and psychological stressors (Brunton & Russell 2010, Brunton *et al.* 2015), together with lower hippocampal *Nr3c1* and *Nr3c2* gene expression, suggesting impaired glucocorticoid negative feedback control over the HPA axis (Brunton & Russell 2010). Sex differences in prenatal stress outcomes are also frequently reported in the offspring (Brunton & Russell 2010, Brunton *et al.* 2011, Glover & Hill 2012); however the mechanisms that underpin these, especially those involved at earlier developmental stages (e.g. *in utero*), have barely been investigated. For example, it is not known whether the reduction in hippocampal *Nr3c1* and/or *Nr3c2* gene expression reported in adult prenatally stressed rats manifest in fetal life, or whether there are any other changes in the developing HPA axis that can impact its function at maturity. Furthermore, the biological signal(s) that permits psychosocial stress, perceived by the mother, to be recognised by the fetus has not been fully identified (Rakers *et al.* 2017).

One mechanism proposed to mediate fetal programming by maternal stress is exposure to excessive maternal glucocorticoids, following activation of the maternal HPA axis (Barbazanges *et al.* 1996, Cottrell & Seckl 2009, Wyrwoll & Holmes 2012, Reynolds 2013). Glucocorticoids play a role in normal fetal growth and development, especially during the third trimester, when a glucocorticoid surge drives the maturation of tissues before birth (Fowden *et al.* 1998). However, excess glucocorticoid signalling can impede fetal growth and development, especially at a time when the brain is vulnerable to changes (Moisiadis & Matthews 2014), and impact placental structure and function (Burton *et al.* 2016). Despite this longstanding hypothesis, a clear relationship between maternal stress, maternal glucocorticoid concentrations and fetal glucocorticoid concentrations has not been convincingly demonstrated in the human or animal literature (Zijlmans *et al.* 2015, Glover *et al.* 2018, Valsamakis *et al.* 2020). Indeed, while repeated stress increases maternal corticosterone concentrations in rats (Ward & Weisz 1984, Brunton & Russell 2010), fetal plasma corticosterone concentrations do not increase in parallel and are similar to levels measured in fetuses from unstressed pregnancies (Ward & Weisz 1984). It is not known whether repeated maternal social stress affects the relationship between maternal and fetal glucocorticoids and no studies have quantified fetal corticosterone and 11-dehydrocorticosterone (11-DHC) concentrations concurrently in response to repeated maternal stress.

Moreover, during pregnancy, several mechanisms exist to protect the developing fetus from exposure to excessive levels of maternal glucocorticoids, further complicating the ‘glucocorticoid hypothesis’. The first is attenuated maternal HPA axis responses to stress during pregnancy. Pregnant rats show significantly lower ACTH and corticosterone secretion in response to acute stressors compared with non-pregnant females (Neumann *et al.* 1998, Johnstone *et al.* 2000, Brunton *et al.* 2009), reducing the pool of glucocorticoids that may potentially be transmitted to the fetus (Brunton *et al.* 2005, Brunton *et al.* 2006, Brunton *et al.* 2009). This is also the case for repeated social stress (Brunton & Russell 2010); however, it is not known whether the stress-induced increase in maternal glucocorticoid secretion, although reduced, is of sufficient magnitude to influence glucocorticoid concentrations in fetal circulation.

The second protective mechanism lies in the placenta, the interface that controls the maternal–fetal exchange of substances. The rat placenta can be divided into two zones: the junctional zone, which contains spongiotrophoblasts, glycogen cells and secondary trophoblast giant cells, where many steroid and peptide hormones are produced, and the labyrinth zone, containing fetal trophoblast cells and capillaries, where most of the maternal–fetal exchange occurs (de Rijk *et al.* 2002). The placenta expresses the enzyme HSD11B2, which in contrast to HSD11B1, catalyses the conversion of active corticosterone (or cortisol in humans) into inert 11-DHC (or cortisone in humans), regulating fetal glucocorticoid exposure (Wyrwoll *et al.* 2011). This enzyme acts as a protective barrier, minimising exposure of the fetuses to excessive levels of maternal glucocorticoids. Indeed, the offspring of rats administered HSD11B2 inhibitors during pregnancy and those of *Hsd11b2* knockout mice display phenotypes reminiscent of those observed in prenatally stressed offspring, including heightened anxiety-like behaviour and HPA axis hyperactivity (Welberg *et al.* 2000, Holmes *et al.* 2006). It has been proposed that maternal stress may lead to the down-regulation of placental HSD11B2; however to date, there is no consensus as to whether maternal stress increases or decreases its expression or activity (Welberg *et al.* 2005, Mairesse *et al.* 2007, Jensen Peña *et al.* 2012, Cuffe *et al.* 2012, Gross *et al.* 2018). On the other hand, the administration of synthetic glucocorticoids upregulates placental *Hsd11b2* and HSD11B2 expression and activity (Ma *et al.* 2003, van Beek *et al.* 2004), perhaps providing a mechanism through which fetal exposure to glucocorticoids can be limited. Moreover, the placenta itself expresses NR3C1 and is responsive to glucocorticoids, which can alter perfusion and nutrient transfer (Fowden & Forhead 2015).

The aim of this study was to investigate the impact of repeated maternal social stress on the regulation of glucocorticoids in the maternal, placental and fetal compartments to better understand whether maternal glucocorticoids are directly involved in fetal programming the offspring. First, we characterised changes in the maternal HPA axis regulation following repeated social stress. Next, we determined whether any changes in corticosterone concentrations in the mother’s circulation are paralleled in the fetus. Finally, we investigated the impact of maternal stress on the expression of genes known to regulate glucocorticoid availability and action in the placenta and fetal brain.

## Materials and methods

### Animals

Female Sprague–Dawley rats (225–250 g on arrival) were purchased from Charles River (Margate, Kent, UK) and maintained on a 07:00–19:00 h light–darkness cycle, under controlled temperature and humidity. After ≥1 week of acclimatisation, female rats were housed overnight with a sexually experienced male. Mating was confirmed by the finding of a semen plug the following morning and this was designated gestational day 1 (GD 1). Two separate cohorts of females underwent mating 1 week apart to generate lactating dams (for use as ‘residents’ in the resident-intruder test) and pregnant experimental rats (‘intruders’ and non-stressed controls). All breeding females were fed a 50:50 mixture of 14 and 19% protein diet *ad libitum* (Teklan, Harlan Laboratories, UK) throughout pregnancy and lactation and had free access to drinking water. Female rats were group housed (4–6/cage) prior to and after mating, until GD16, after which time all rats were housed individually. All animal experiments reported here were approved by the University of Edinburgh Animal Welfare and Ethical Review Body and performed in accordance with the UK Animals (Scientific Procedures) Act 1986.

### Induction of social stress

A modified resident-intruder paradigm was used to induce social stress as previously described (Brunton & Russell 2010). This paradigm has previously been characterised as a relevant stressor for female rats (Neumann *et al.* 2001, Brunton & Russell 2010). Experimental pregnant dams (‘intruders’; *n* = 7) were transferred to the home-cage of an unfamiliar lactating dam (‘residents’; days 1–7 of lactation) for 10 min/day from GD16 to GD20, between 10:00 and 14:00 h. On GD16–19, experimental pregnant dams were returned to their cages immediately after the social stress. Non-stressed pregnant controls (*n* = 7) remained individually housed from GD16 to GD20 and were undisturbed except for daily weighing. On GD20, pregnant control and stressed dams were killed and tissues collected (see later). Control dams were undisturbed prior to being killed, while stressed dams were killed immediately after the final 10 min bout of social stress on GD20.

### Tissue collection

Pregnant dams were killed by conscious decapitation on GD20. Trunk blood was collected into chilled tubes containing 5% (w/v) EDTA. Fetuses and placenta were rapidly removed and the sex of the feto-placental unit was determined by examining anogenital distance. Trunk blood from decapitated fetuses was collected using EDTA-coated capillary tubes and pooled by sex (from 3 to 10 fetuses depending on litter size and the sex ratio of the litter) and litter. Maternal and fetal brains, placenta and fetal liver were rapidly collected and frozen on dry ice, then stored at −80°C until further processing. Blood was centrifuged; then plasma was separated and stored at −20°C until further analyses.

### Liquid chromatography tandem mass spectrometry

Liquid chromatography-tandem mass spectrometry (LC-MS/MS) quantification of steroids was carried out as described previously (Sze *et al.* 2018, Sze & Brunton 2021), with a modified method to quantify total corticosterone and 11-DHC. Briefly, C18 solid phase extraction was performed on 50 mg of fetal liver, 1/8th of a placenta (‘pie slice’ containing both junctional and labyrinth zones), 1 hemisphere of a fetal brain or plasma (diluted 1:100). Plasma was pooled by sex for each litter, while one male and one female placenta, fetal liver or fetal brain from each litter were used for analysis. All samples were processed with corticosterone-d4 (#802905; Sigma) as an internal standard, alongside calibration standard solutions of 25,000, 10,000, 4000, 1600, 640, 256, 102.4 pg/mL corticosterone and 11-DHC (#Q1550-000 and #Q3690-000; both Steraloids Inc., RI, USA), under the same conditions as the samples (Supplementary Materials, see section on supplementary materials given at the end of this article). Extracted standards and samples then underwent C18 reverse phase liquid chromatography, followed by positive electrospray ion trap mass spectrometry (Suppl. Info). Calibration curves were constructed using the ratio of the peak areas of corticosterone or 11-DHC to the peak areas of corticosterone-d4 obtained from the LC/MS analyses (Supplementary Materials). Concentrations of samples were extrapolated, and converted to ng/mL (for plasma) or normalised to the wet weight of the tissues (ng/g for brain, liver and placenta).

### Adrenocorticotropic hormone radioimmunoassay

Maternal plasma adrenocorticotropic hormone (ACTH) concentrations were measured in duplicate samples using an ACTH Double Antibody RIA Kit (MP Biomedicals, Eschwege, Germany; #07106102) in accordance with the manufacturer’s instructions.

### *In situ* hybridisation

*In situ* hybridisation (ISH) was used to investigate the expression of genes known to regulate or modulate glucocorticoid activity and signalling in the maternal brain, maternal pituitary gland, placenta and fetal brain. ISH was performed on 15 μm coronally sectioned maternal and fetal brains and midline-sections of placenta, using oligonucleotide probes (Supplementary Table 1A) for arginine vasopressin (*Avp*) and pro-opiomelanocortin (*Pomc*) or riboprobes (Supplementary Table 1B) for corticotropin-releasing hormone (*Crh*), glucocorticoid receptor (*Nr3c1*), mineralocorticoid receptor (*Nr3c2*), 11β-hydroxysteroid dehydrogenase type 1 (*Hsd11b1*) and type 2 (*Hsd11b2*), FK506-binding protein 51 (*Fkbp51*) and 52 (*Fkbp52*), as previously described (Brunton *et al.* 2005, Brunton *et al.* 2009). One male and one female placenta/fetal brain from each litter were used for *in situ* hybridisation. For further details, refer to the Supplementary Materials.

### Western blotting

Western blotting was used to quantify placental HSD11B2 expression (one male and one female placenta/litter were used; *n* =  7/group). Briefly, 1/8th of a placenta containing both junctional and labyrinth zones was homogenised, and 50 µg protein/sample was electrophoresed on a gel and transferred onto a PVDF membrane. Membranes were blocked and incubated with a primary antibody targeting HSD11B2 (Abcam, #ab80317; 1:250), followed by a fluorescent secondary anti-rabbit IgG. After visualisation, the membrane was stripped and incubated again with a primary antibody targeting β-actin (Sigma, #A5411; 1:50,000) and a fluorescent secondary anti-mouse IgG and visualised again. Band intensity was quantified on ImageJ and HSD11B2 band intensity was normalised against β-actin band intensity to obtain HSD11B2 expression for each placenta sample. For further details, refer to the Supplementary Materials.

### Data analysis and statistics

**Figure 1:**
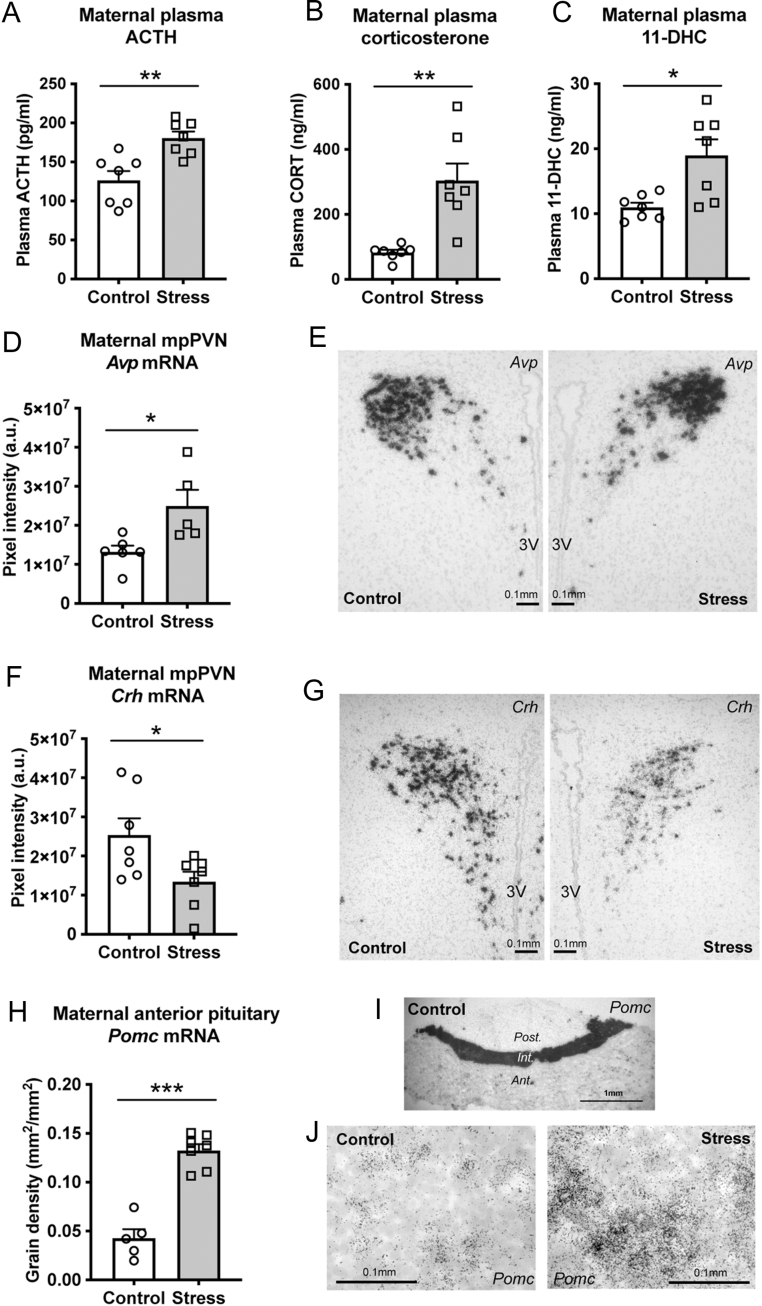
Effect of maternal social stress on maternal HPA axis activity. Repeated social stress for 5 days resulted in an increase in (A) ACTH, (B) corticosterone and (C) 11-dehydrocorticosterone (11-DHC) concentrations in the maternal plasma. In the medial parvocellular region of the paraventricular nucleus (mpPVN), stressed dams displayed greater expression of arginine vasopressin (*Avp*) mRNA (D, E) and lower expression of (F, G) corticotropin-releasing factor (*Crh*) mRNA. In the anterior pituitary gland, stressed dams had greater pro-opiomelanocortin (*Pomc*) mRNA expression compared to non-stressed controls (H, J). Representative images show clusters of silver grains indicating (E) *Avp* and (G) *Crh* mRNA hybridisation in the maternal mpPVN and (I) *Pomc* mRNA expression in the pituitary gland. (J) Representative high-power image of *Pomc* expression in the anterior lobe of the pituitary gland. *P < 0.05, **P < 0.01, ***P < 0.001. Individual data points (*n*  = 7/group) overlay bars representing group means + s.e.m. Average pixel intensity is presented for (D) *Avp* and (F) *Crh* due to the greater intensity of silver grains. (H) *Pomc* was quantified using grain density (mm^2^/mm^2^). 3V, third ventricle; Ant., anterior; Int., intermediate; Post., posterior lobes of the pituitary gland.

**Figure 2:**
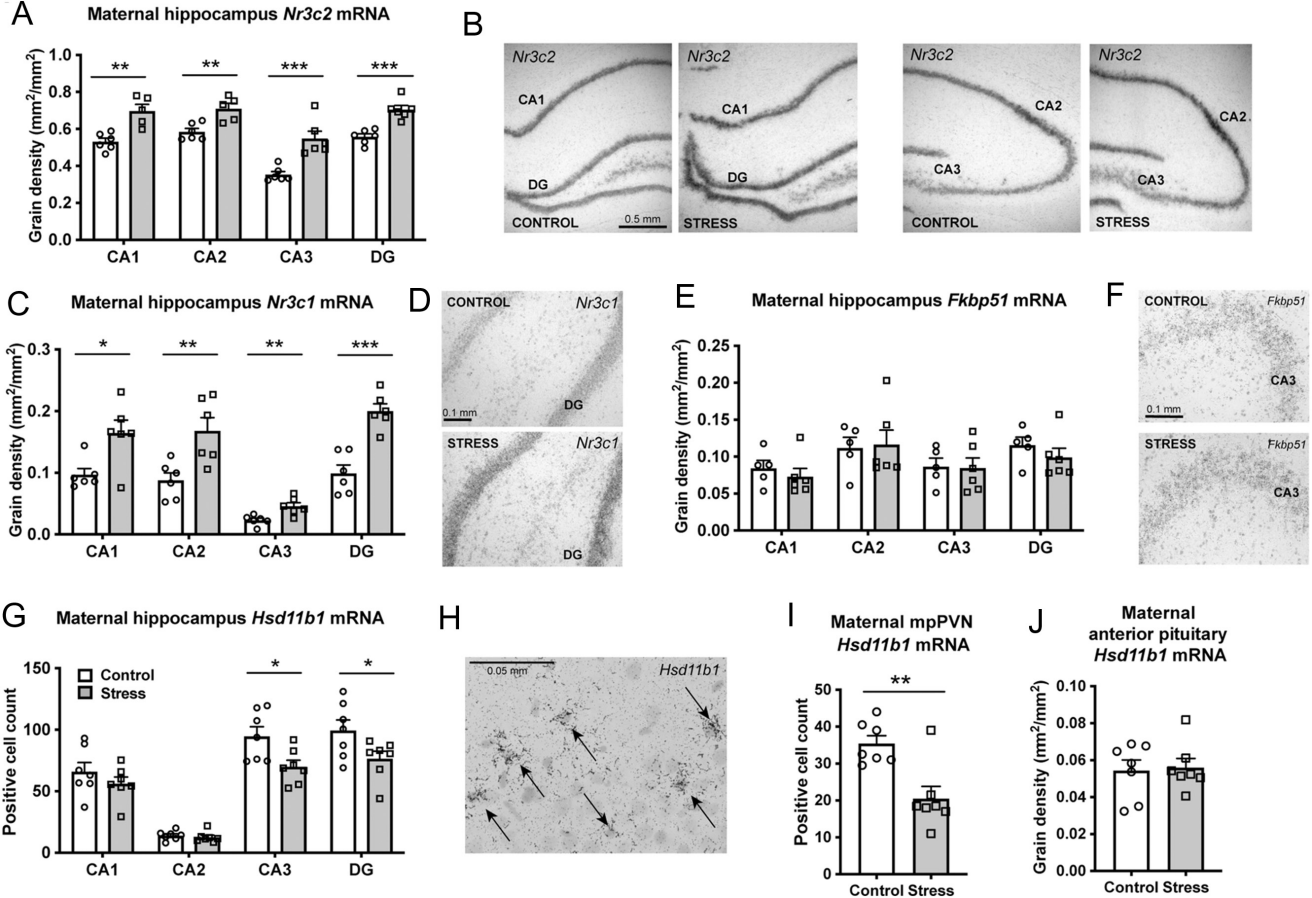
Effect of maternal social stress on maternal HPA axis regulation. Gene expression for (A) mineralocorticoid receptor (*Nr3c2*) and (C) glucocorticoid receptor (*Nr3c1*) was greater in each of the hippocampal subfields in the stressed dams (grey bars) compared to the non-stressed control dams (white bars). Representative images of the four regions of the hippocampus expressing (B) *Nr3c2* and (D) *Nr3c1* mRNA expression in the dentate gyrus (DG). (E, F) *Fkbp51* expression did not differ between stressed and control dams in any of the hippocampal regions. 11β-hydroxysteroid dehydrogenase type 1 (*Hsd11b1*) mRNA expression was lower in (G) the CA3 and DG region of the hippocampus and (I) medial parvocellular paraventricular nucleus (mpPVN) of the stressed dams, but not altered in (J) the anterior pituitary gland. (H) Representative photomicrograph shows an example of cells positively expressing *Hsd11b1* mRNA (indicated by arrows) in the hippocampus. *P < 0.05, **P < 0.01, ***P < 0.001. Individual data points (*n*  = 6–7/group) overlay bars representing group means + s.e.m. Grain density (mm^2^/mm^2^) is presented in each case, except for *Hsd11b1* in the maternal hippocampus (G) and mpPVN (I) where the number of positive cells was counted.

**Figure 3:**
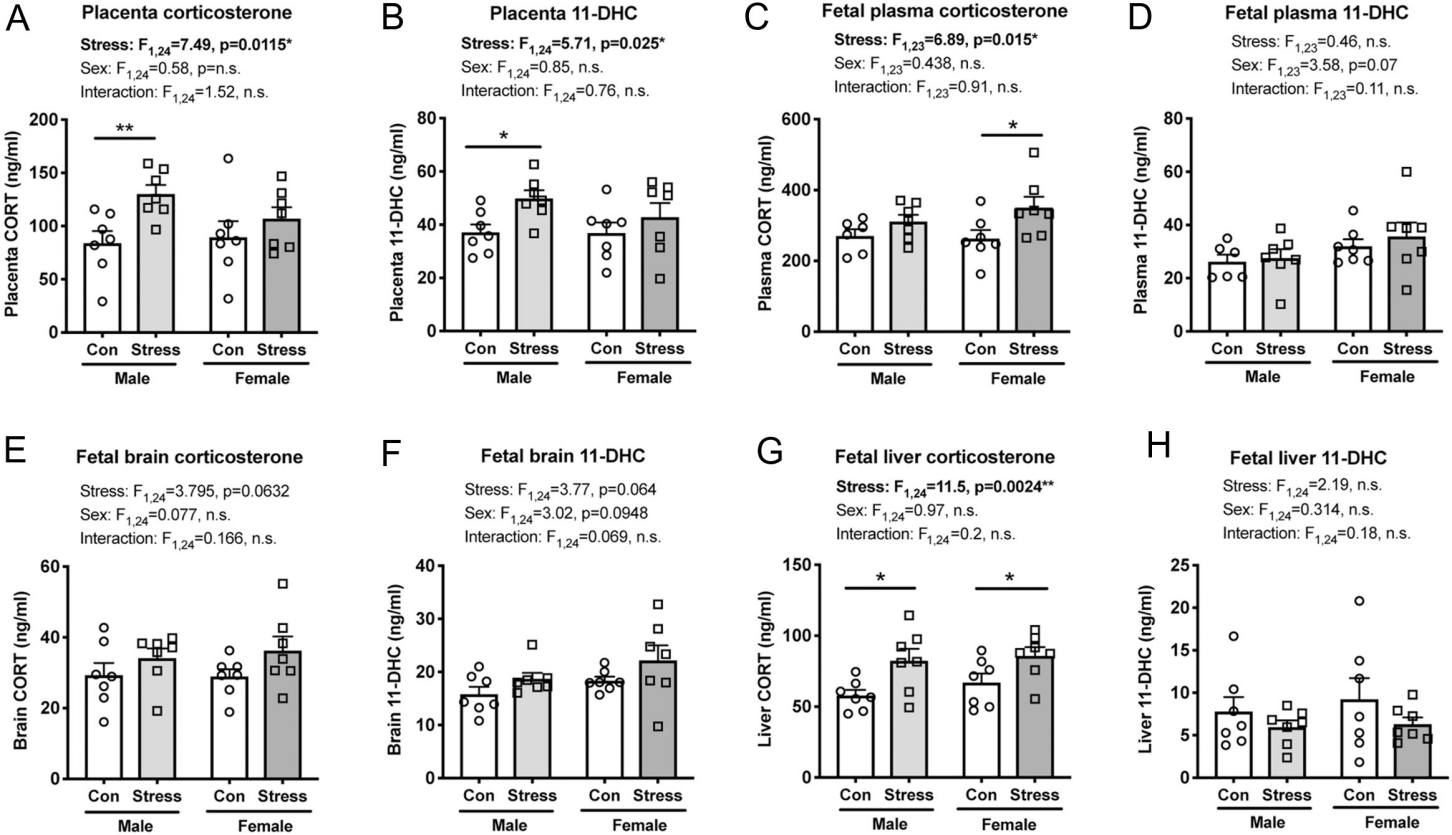
Effect of maternal social stress on corticosterone and 11-DHC concentrations in the placenta and fetus. Both (A) corticosterone and (B) 11-DHC concentrations were greater in the placenta of stressed male fetuses than in the placenta of control male fetuses, with no significant differences in the female fetuses. (C) Plasma corticosterone concentrations were greater in the stressed female fetuses compared to control female fetuses, with no differences in the male fetuses. (D) Plasma 11-dehydrocorticosterone (11-DHC) concentrations were not altered by stress in either sex. In the fetal brain, no differences were detected in (E) corticosterone or (F) 11-DHC concentrations in either sex. (G) Hepatic corticosterone concentrations were greater in both male and female stressed fetuses compared to their respective controls, while (H) 11-DHC did not differ between any of the groups. *P < 0.05, **P < 0.01, ***P < 0.001. Individual data points (*n*  = 6–7/group) overlay bars representing group means + s.e.m.

**Figure 4:**
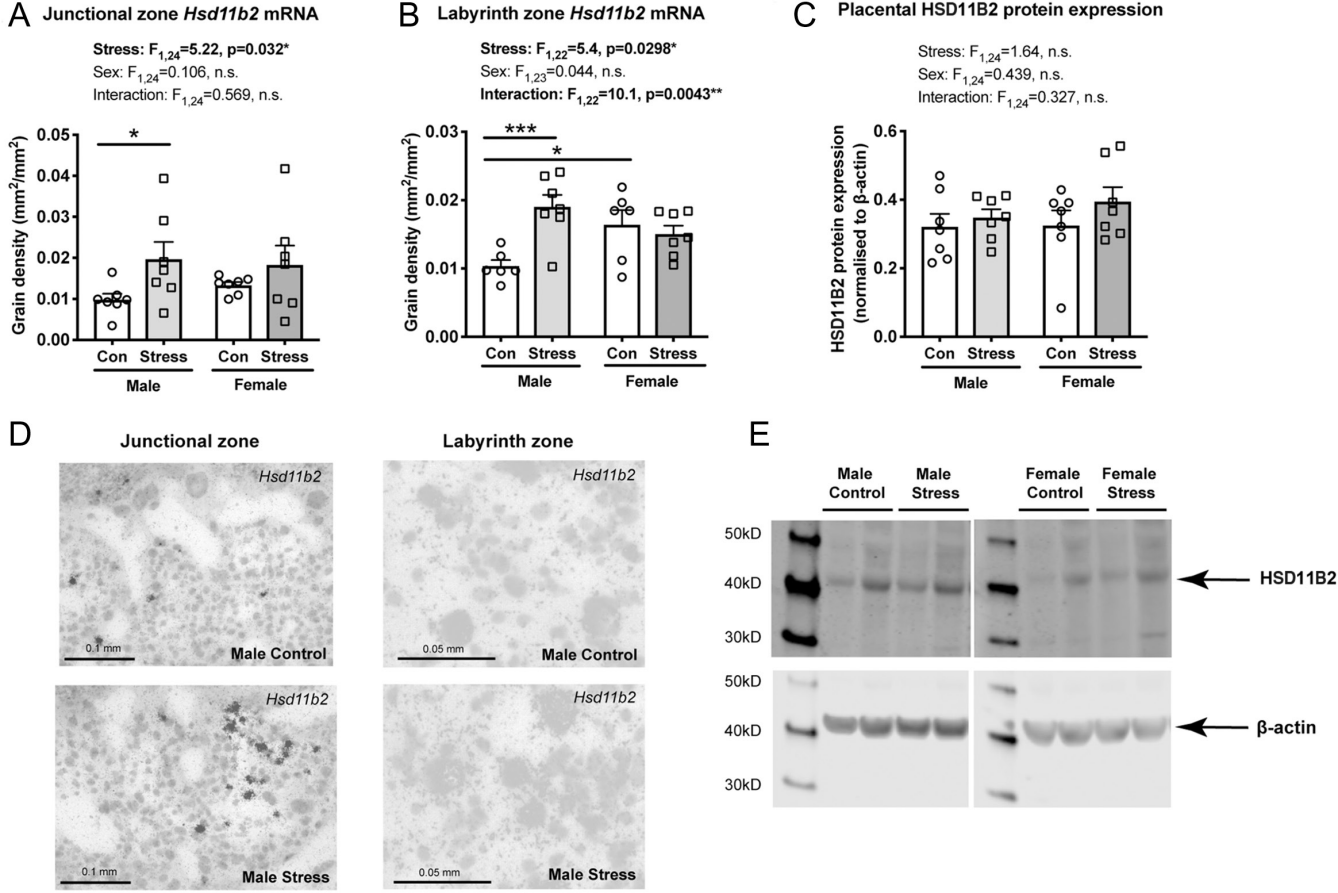
Effect of maternal social stress on gene and protein expression for 11β-hydroxysteroid dehydrogenase type 2 in the placenta. 11β-hydroxysteroid dehydrogenase type 2 (*Hsd11b2*) mRNA expression in both the placental (A, D – left) junctional and (B, D – right) labyrinth zones from stressed males was greater compared to control male placentae. While no differences in *Hsd11b2* expression were observed between control and stressed female placentae in either zone (A, B), female control placenta displayed significantly greater *Hsd11b2* mRNA expression compared to male controls in the labyrinth zone (B). There were no effects of stress or sex on placental 11β-HSD2 protein expression (C). Representative Western blots of 11β-HSD2 (E – top panel) and β-actin (E – bottom panel), after stripping and re-probing. *P < 0.05, ***P < 0.001. Individual data points (*n* = 6–7/group) overlay bars representing group means + s.e.m.

**Figure 5:**
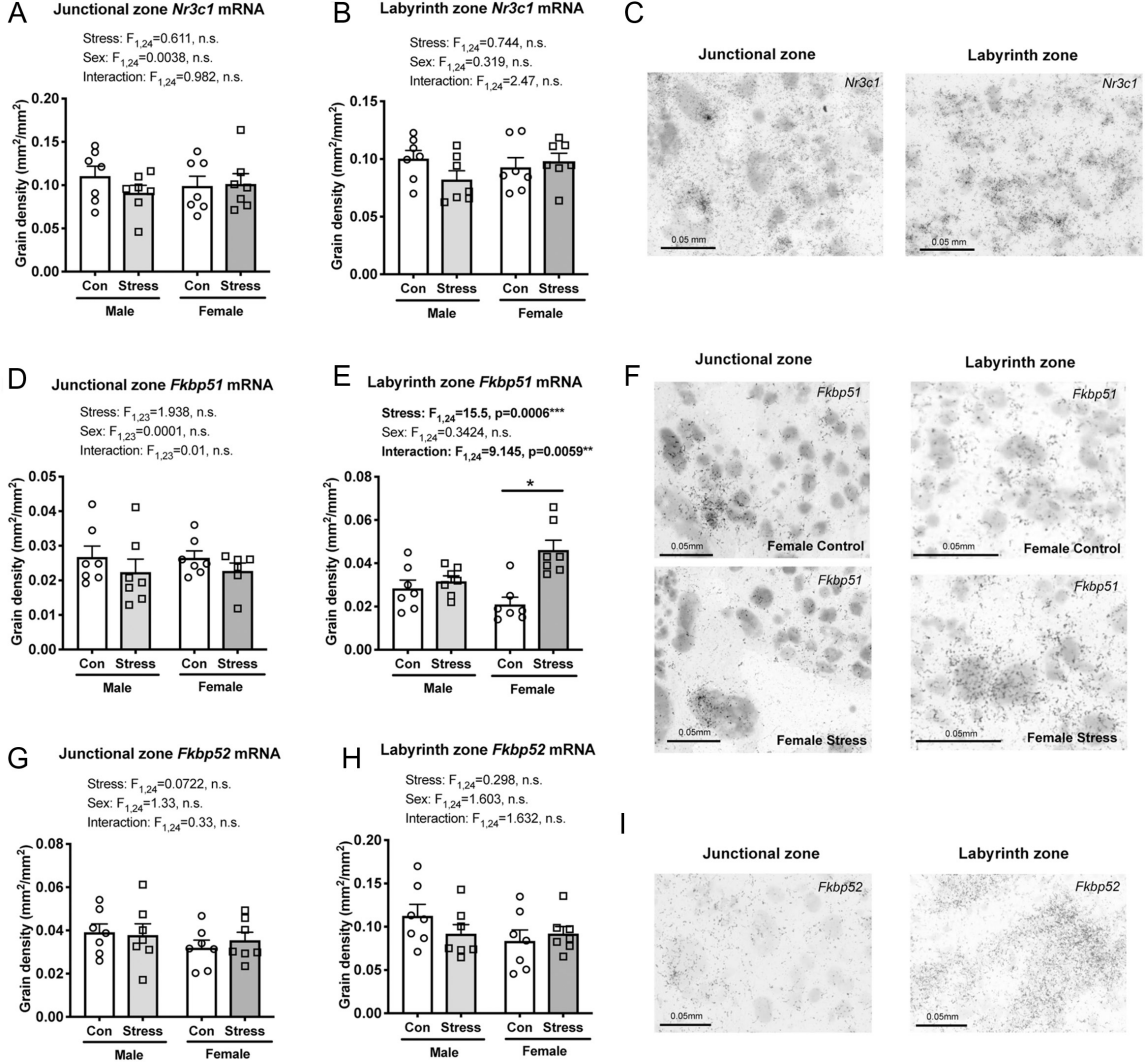
Effect of maternal social stress on glucocorticorticoid regulatory mechanisms in the placenta. No differences were observed for glucocorticoid receptor (*Nr3c1*) expression between any of the groups in either region of the placenta (A, B). (C) Representative photomicrograph of *Nr3c1* expression in the placenta. There was no difference in *Fkbp51* expression between groups in the junctional zone (D, F – left); however, *Fkbp51* expression in the labyrinth zone was significantly greater in stressed female placenta compared with control female placenta (E, F – right). No differences were detected in *Fkbp52* mRNA expression between any of the groups in either the (G) junctional or (H) labyrinth zone of the placenta. (I) Representative photomicrograph of *Fkbp52* expression in the junctional (left) and labyrinth zone (right) of the placenta. *P < 0.05, ***P < 0.001. Grain density (mm^2^/mm^2^) is presented in each case. Individual data points (*n*  = 6–7/group) overlay bars representing group means + s.e.m.

Statistical analyses were performed using PRISM 6.0 (GraphPad Software Inc.). *T*-test with Welch’s correction was used for comparisons between control and stressed maternal groups (Figs. 1 and 2). Two-way ANOVA (with stress and sex as main factors) were used for comparisons in the placenta and fetuses, with Fisher's least significance difference test as the* post-hoc* test (Figs. 3-5). *P* < 0.05 was considered statistically significant in each case.

## Results

### Effect of repeated social stress on maternal body weight and litter size

Social stress exposure did not significantly affect the body weight gain of the dams between days 16 and 20 of pregnancy (% increase in body weight was 13.4 ± 1.2% in the controls and 12.3 ± 1.3% in the stressed dams; *t* = 0.63, *P* = 0.54). There were no significant differences in the total number of fetuses (control: 14 ± 0.5, stress: 15 ± 0.4, *t* = 1.71, *P* = 0.11) or the male:female ratio (control: 1.3 ± 0.2, stress: 0.8 ± 0.2, *t* = 1.625, *P* = 0.13) of the litters from control and stressed dams.

## Effect of repeated social stress on maternal HPA axis activity and regulation

Following the final bout of social stress on GD20, plasma concentrations of ACTH (1.4-fold; *P* = 0.0034, *t* = 3.75; Fig. 1A), corticosterone (3.7-fold; *P* = 0.0052, *t* = 4.18; Fig. 1B) and 11-DHC (1.7-fold; *P* = 0.0174, *t* = 3.10; Fig. 1C) were significantly greater in the stressed dams compared to the non-stressed control dams.

Repeated social stress also resulted in significantly greater *Avp* expression (*P* = 0.044, *t* = 2.64; Fig. 1D-E), but lower *Crh* expression (*P* = 0.0373, *t* = 2.41; Fig. 1F-G) in the mpPVN compared with non-stressed controls. Gene expression of the ACTH precursor, *Pomc*, was markedly greater in the anterior pituitary gland of stressed dams compared to control dams (*P* < 0.0001, *t* = 7.91; Fig. 1H-J).

Stressed dams also had greater *Nr3c2* (Fig. 2A-B) and *Nr3c1* expression (Fig. 2C-D) in all four regions of the hippocampus (*Nr3c2*: CA1: *P* = 0.0014, *t* = 4.56; CA2: *P* = 0.0021, *t* = 4.26; CA3: *P* = 0.00024, *t* = 5.57; dentate gyrus (DG): *P* = 0.04, *t* = 2.36; *Nr3c1*: CA1: *P* = 0.014, *t* = 2.96; CA2: *P* = 0.0082, *t* = 3.29; CA3: *P* = 0.007, *t* = 3.38; DG: *P* = 0.0002, *t* = 5.66). Gene expression for *Fkbp51* did not differ between the stressed and control dams in any of the hippocampal subfields (Fig. 2E-F).

We also investigated whether the ability to modulate local glucocorticoid concentrations in the hippocampus was affected by repeated social stress by quantifying changes in the expression of *Hsd11b1*, which converts inert 11-DHC into corticosterone. *Hsd11b1* expression in the CA3 and DG of the hippocampus was significantly lower in stressed dams compared to control dams (CA3: *P* = 0.021, *t* = 2.66; DG: *P* = 0.048, *t* = 2.199) on GD20, but no differences were observed in the CA1 and CA2 regions (Fig. 2G-H). The number of *Hsd11b1* expressing cells was also lower in the mpPVN of stressed dams compared to control dams (*P* = 0.0034, *t* = 3.79; Fig. 2I), but no differences were observed in the anterior pituitary gland (Fig. 2J).

### Impact of maternal stress on placental and fetal glucocorticoid concentrations

We next investigated whether the increase in corticosterone and 11-DHC detected in the maternal compartment was also evident in the placenta and the fetus, indicating maternal–fetal transmission.

In the placenta, there was a significant main effect of stress on both placental corticosterone and 11-DHC concentrations. The male placentae from stressed mothers contained significantly greater concentrations of both corticosterone (*P* = 0.0098; Fig. 3A) and 11-DHC (*P* = 0.03; Fig. 3B), compared to the male placentae from control rats; however, this difference was not observed in female placentae (corticosterone: *P* = 0.297, 11-DHC: *P* = 0.294; Fig. 3A-B).

There was a significant main effect of stress on fetal plasma corticosterone concentrations (Fig. 3C). *Post-hoc* comparisons revealed significantly greater plasma corticosterone concentrations in the stressed female fetuses compared with the control females (*P* = 0.017; Fig. 3C), albeit to a lesser extent (1.3-fold) than that observed between the stressed and controls dams (3.7-fold). In contrast, there was no significant difference in plasma corticosterone concentrations between the stressed and control male fetuses (*P* = 0.259; Fig. 3C). Plasma 11-DHC concentrations did not differ between control and stressed fetuses of either sex (Fig. 3D).

In the fetal brain, there were no significant differences in corticosterone (Fig. 3E) or 11-DHC concentrations between any of the groups (Fig. 3F). Whereas in the fetal liver, there was a main effect of stress on corticosterone concentrations (Fig. 3G). Corticosterone concentrations in the liver were significantly greater in stressed fetuses compared to control fetuses, for both males (*P* = 0.012) and females (*P* = 0.048). Hepatic 11-DHC concentrations were not different between the four groups (Fig. 3H).

### Effect of maternal stress on glucocorticoid metabolism and action in the placenta

Next, we quantified gene expression for modulators of glucocorticoid action in the placenta. A main effect of stress was observed for *Hsd11b2* expression in both the junctional and labyrinth zones of the placenta (Fig. 4A, B), with an additional sex × stress interaction detected only in the labyrinth zone (Fig. 4B). *Hsd11b2* expression was significantly greater in stressed male placentae compared to control male placentae in both the junctional (*P* = 0.0419; Fig. 4A, D) and labyrinth zones (*P* = 0.0008; Fig. 4B, D); whereas in females, no significant differences in *Hsd11b2* expression were observed (*P* = 0.29 for junctional zone, *P* = 0.55 for labyrinth zone). Additionally, in the labyrinth zone, control females were found to have significantly greater *Hsd11b2* expression compared to control males (*P* = 0.0157); however, this sex difference was not observed in the stressed groups (*P* = 0.0774; Fig. 4B). In contrast to the gene expression data, there was no significant effect of maternal social stress on placental HSD11B2 in either sex (Fig. 4C, E).

There were no differences in placental *Nr3c1* expression between any of the four groups in either the junctional (Fig. 5A, C) or labyrinth zones (Fig. 5B, C).

There was a main effect of stress and a stress × sex interaction for *Fkbp51* gene expression in the labyrinth zone (Fig. 5E), but not in the junctional zone (Fig. 5D) of the placenta. Stressed female placentae had significantly greater *Fkbp51* expression than control females (*P* < 0.0001) and stressed males (*P* = 0.0094) in the labyrinth zone (Fig. 5E, F). Gene expression for *Fkbp52* did not differ across the four groups in either the junctional (Fig. 5G, I) or labyrinth zone (Fig. 5H, I).

### Effect of maternal stress on glucocorticoid metabolism and action in the fetal hippocampus

**Figure 6:**
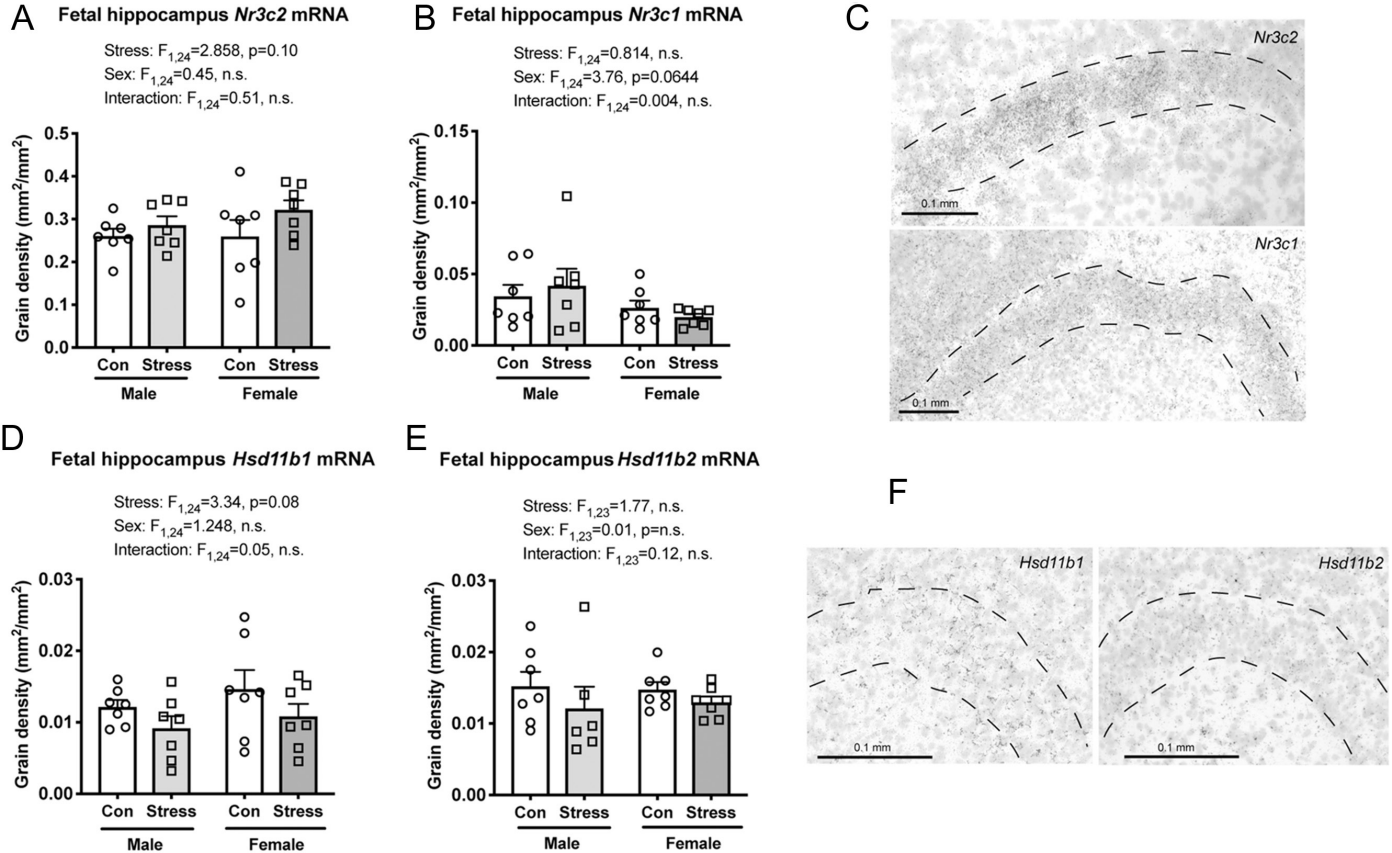
Effects of maternal social stress on glucocorticoid regulatory mechanisms in the fetal hippocampus. No differences in mRNA expression for (A) mineralocorticoid receptor (*Nr3c2*), (B) glucocorticoid receptor (*Nr3c1*), (D) 11β-hydroxysteroid dehydrogenase type 1 (*Hsd11b1*) or (E) 11β-hydroxysteroid dehydrogenase type 2 (*Hsd11b2*) were observed in the hippocampus between any of the groups. Representative photomicrographs show examples of hybridisation for (C) *Nr3c2* (top) and *Nr3c1* (bottom), and (F) *Hsd11b1* (left) and *Hsd11b2* (right). Grain density (mm^2^/mm^2^) is presented in each case. Individual data points (*n*  = 6–7/group) overlay bars representing group means + s.e.m.

Finally, we sought to investigate whether maternal stress induced any other changes in genes that modulate glucocorticoid activity or action specifically in the fetal hippocampus. There were no differences in the expression of either *Nr3c2* (Fig. 6A, C) or *Nr3c1* (Fig. 6B,  C), nor were there any differences in *Hsd11b1* (Fig. 6D, F) or *Hsd11b2* (Fig. 6E, F) expression between the four groups.

## Discussion

Here we show that repeated social stress activates the HPA axis in late pregnant dams. Despite the large increase in corticosterone secretion in the pregnant dam, this evidently did not access the fetal compartment – only a modest increase in corticosterone was observed in the circulation of female fetuses, with no change in the male fetuses. Importantly, there was no stress-induced changes in corticosterone concentrations in the fetal brain of either sex. Sex-dependent changes were observed in the expression of genes involved in regulating glucocorticoid availability and action in the placenta. It is therefore expected that any role for maternal glucocorticoids in fetal programming the offspring’s brain and behaviour occurs via indirect actions, possibly through inducing changes in the placenta, rather than via direct crossover into the fetal circulation and subsequently accessing the fetal brain.

### Maternal HPA axis

The maternal HPA axis was activated by social stress, reflected by the increased secretion of ACTH and corticosterone and an up-regulation of *Pomc* expression in the anterior pituitary gland, probably serving to replenish ACTH stores following its secretion (Zelena *et al.* 2003). Given corticosterone secretion increases rapidly in response to stress, the increase measured here likely reflects the stress experienced during the final social stress bout. Nevertheless, the increase in ACTH and corticosterone secretion indicates the maternal HPA axis response to social stress persists even after repeated exposure, consistent with our previous findings using this stress paradigm, where we demonstrated that although corticosterone secretory responses are attenuated in late pregnant rats compared with virgin females, they are markedly greater than baseline and those measured in non-stressed pregnant rats (Brunton & Russell 2010). Circulatory 11-DHC concentrations were also elevated in the stressed dams, indicating the protective mechanism responsible for inactivating maternal corticosterone (e.g. placental HSD11B2) was functional following stress. We also detected altered gene expression in the maternal mpPVN. *Avp* expression was significantly greater and *Crh* expression was lower in stressed dams compared with non-stressed dams. These changes are likely a result of repeated social stress, as they mirror the changes observed following repeated stress in non-pregnant rats (Ma *et al.* 1997). While acute stress induces upregulation of *Crh* gene transcription (which typically predominates), in contrast, following repeated homotypic stressors, habituation occurs in the *Crh* response resulting in a shift in the hypothalamic driver of ACTH secretion in favour of *Avp*, which helps maintain HPA axis reactivity (Scaccianoce *et al.* 1991, de Goeij *et al.* 1992, Ma *et al.* 1997). Our data suggest that the HPA axis response, and critically corticosterone secretion, to repeated social stress is sustained in late pregnant rats, with the key ACTH secretagogue being *Avp*, rather than *Crh*. This is of interest as a lack of *Avp*, rather than *Crh*, secretion into the portal blood evidently underpins HPA axis hyporesponsiveness to acute stress in late pregnancy (Ma *et al.* 2005). Hence, the greater activation of *Avp*-synthesising neurones in the mpPVN observed here may indicate this protective mechanism that restrains stress-induced HPA axis activation is disrupted under conditions of repeated stress exposure.

Changes related to glucocorticoid negative feedback control of the maternal HPA axis were also observed. Hippocampal *Nr3c1* and *Nr3c2* expression in the pregnant dams was significantly upregulated following repeated social stress, with no change in expression of the Nr3c1 co-chaperone, *Fkbp51*. Exposure of these receptors to increased levels of the ligand through exogenous administration or chronic stress is typically expected to lead to down-regulation of *Nr3c1* and *Nr3c2* transcription, at least in male rodents (Gómez *et al.* 1996, Paskitti *et al.* 2000, Hügin-Flores *et al.* 2004); however, in late pregnant rats, the opposite was observed. This may be a compensatory mechanism serving to enhance negative feedback control over the HPA axis and dampen corticosterone responses given attenuated HPA axis responses to stress are well established in late pregnancy and are considered protective (Brunton *et al.* 2008, Brunton & Russell 2011, Russell & Brunton 2019). Indeed, basal expression of *Nr3c1* is up-regulated in the dentate gyrus in late pregnancy (Johnstone *et al.* 2000). In contrast, *Hsd11b1* expression in the mpPVN and hippocampal CA3 and DG regions was lower in the stressed dams. In late pregnancy, HSD11B1 activity is increased in the PVN and anterior pituitary gland (though not the hippocampus), where it is expected to enhance local intracellular glucocorticoid concentrations and promote negative feedback control of the HPA axis (Johnstone *et al.* 2000). The promoter region of the *Hsd11b1* gene possesses a glucocorticoid-response element (Moisan *et al.* 1992a, Yang *et al.* 2007) and glucocorticoids acting via Nr3c1 up-regulate *Hsd11b1* promoter activity and gene transcription (Low *et al.* 1994, Yang *et al.* 2007), so here the finding of lower *Hsd11b1* expression in the stressed dams was unexpected. However, alterations in *Hsd11b1* expression may provide a mechanism through which the exposure of glucocorticoid-sensitive neurones, such as those in the hippocampus and PVN, to corticosterone can be regulated locally to ensure optimal exposure under conditions of repeated stress and elevated corticosterone levels.

Overall, these changes suggest that regulation of the maternal HPA axis following chronic stress as compared to single acute stressors during pregnancy is complex; however, the maternal HPA axis is able to mount a significant corticosterone response and glucocorticoid negative feedback mechanisms do not appear impaired.

### Changes in the placenta and glucocorticoid transfer

The fundamental basis of the glucocorticoid programming hypothesis centres around the assumption that circulating glucocorticoid concentrations are considerably lower in the fetus than in the mother (Chapman *et al.* 2013). Excess glucocorticoids, which are small lipophilic molecules, would therefore have the tendency to cross the placenta via simple diffusion unless inactivated by placental HSD11B2. Here, contrary to these assumptions, absolute concentrations of both fetal circulating corticosterone and 11-DHC were greater than circulating maternal concentrations. Furthermore, following maternal stress, corticosterone concentrations were unchanged in the male fetuses and only 1.3-fold greater than non-stressed controls in the female fetuses, which is a modest increase compared with the 3.7-fold increase measured in the mothers. These measurements are consistent with previous studies in rats using radioimmunoassays, where increases in plasma corticosterone concentrations in the fetuses following maternal stress were of far lower magnitude than those observed in the pregnant dam (Ward & Weisz 1984, Takahashi *et al.* 1998, Williams *et al.* 1999, Bingham *et al.* 2013, Scott *et al.* 2020).

The sex differences observed in fetal plasma corticosterone concentrations also corroborate findings in mice, where a small increase in corticosterone levels is observed in the female, but not in the male fetuses following maternal restraint stress (Montano *et al.* 1993). This sex difference may suggest placental transport of corticosterone from the maternal blood is greater in the female than in the male fetuses, especially given stress up-regulated placental *Hsd11b2* in the male, but not the female fetuses, which would be expected to provide a stronger barrier against corticosterone transfer to male fetuses, resulting in an increase in circulating corticosterone only in the female fetuses. However, in view of the finding that *Hsd11b2* expression was already significantly greater in the placental labyrinth zone (the major site of feto-maternal exchange) of female fetuses compared with male fetuses under non-stress conditions (such that there was no sex difference in the fetuses of stressed dams) and that protein expression of Hsd11b2 was not different between sexes, this seems unlikely. Instead, it may be that the small increase in circulatory corticosterone concentrations in female fetuses following maternal stress arises from increased secretion by the fetuses’ own adrenal glands, which in rats are capable of secreting corticosterone from GD 16 (Boudouresque *et al.* 1988). The fetal HPA axis responds to maternal stress during pregnancy (Williams *et al.* 1999, Ohkawa *et al.* 1991); however, the mechanisms involved in the fetal perception of maternal stress and fetal HPA axis activation are unclear, though may involve hypoxia due to reduced uterine blood flow or increased oxidative stress (Morishima *et al.* 1979, Ohkawa *et al.* 1991, Scott *et al.* 2020).

Comparable sex differences have been reported in human placenta, with *Hsd11b2* more highly expressed in placentae of females than males (Mericq *et al.* 2009), while maternal stress is associated with increased *Hsd11b2* expression in male placentae and reduced expression in female placentae (Mina *et al.* 2015). Here, stress-induced up-regulation of placental *Hsd11b2* in the male fetuses may be driven by increased placental corticosterone concentrations (van Beek *et al.* 2004), a finding observed in males, but not in females. Placental HSD11B2 protein expression was not correlated with *Hsd11b2* gene expression. This is not entirely surprising since it is accepted that mRNA transcript abundance only partially predicts protein abundance, for a range of reasons, e.g. post-transcriptional regulation, different production, degradation and stability rates between mRNA and protein (Vogel & Marcotte 2012). Nevertheless, our data do not support a role for maternal stress in down-regulating HSD11B2, rather the data suggest that the protective HSD11B2 placental barrier, considered to minimise fetal exposure to maternal glucocorticoids, is not compromised by maternal social stress during pregnancy. Indeed, recent studies using human placenta have questioned the importance of HSD11B2 in protecting the fetus from high maternal glucocorticoid transfer, as less than 10% of labelled cortisol crosses from the maternal to fetal circulation even when HSD11B2 is inhibited (Stirrat *et al.* 2018). These findings suggest that in addition to the well-established inactivation of glucocorticoids by HSD11B2, additional mechanisms may play an important role in regulating glucocorticoid transport, such as placental ATP-binding cassette transporters (Bloise *et al.* 2016), but any potential influences of maternal stress on these mechanisms have not yet been investigated.

Given that glucocorticoids influence placental function (Fowden & Forhead 2015), it is possible that placental HSD11B2 plays a role in regulating local corticosterone levels, which may in turn affect placental functions such as oxygen and/or nutrient transfer. Here, placentae of stressed female, but not male fetuses, displayed increased *Fkbp51* expression in the labyrinth zone. While placental *Nr3c1* expression remained unchanged following stress in both sexes, Nr3c1 signalling within the placenta may be reduced in the stressed females as FKBP51 inhibits NR3C1 action. These data suggest that the male and female placentae may adopt different strategies to mitigate the effects of maternal stress. Several sex differences in the placenta that contribute to prenatal programming have been identified, including glucocorticoid regulation (Schmidt *et al.* 2019), and epigenetic mechanisms (Howerton & Bale 2014). Therefore, it is possible that sex differences in adulthood behavioural outcomes in prenatally stressed rats can be traced back to sexually dimorphic mechanisms in the placenta during development, which remain poorly understood.

### Changes in the fetal brain and liver

Ultimately, the physiological effects of glucocorticoids in the fetus are dependent on their action in target organs. While glucocorticoids are required for normal fetal growth, the expression of their receptors, receptor chaperone proteins and converting enzymes undergo dynamic changes to continually fine-tune the system to ensure appropriate glucocorticoid action in the fetal tissues. In this study, there were no changes in corticosterone or 11-DHC concentrations in the fetal brain of either sex, despite the slightly greater circulating concentrations in the female fetuses. This is the first time corticosterone and 11-DHC have been quantified in the developing rat brain in both sexes, and no sex differences in corticosterone were observed at this stage, contrary to the adult brain when the HPA axis is fully mature (Sze *et al.* 2018). However, there could be regional differences as recently described in the developing mouse brain (Hamden *et al.* 2021). Nevertheless, our current data do not support a role for maternal glucocorticoids exerting direct actions on the fetal brain to exert programming effects.

*Nr3c1* and *Nr3c2* were not altered by stress in the fetal hippocampus; thus, the reduction in receptor gene expression observed in adulthood (Brunton & Russell 2010) likely emerges postnatally. *Hsd11b1* and *Hsd11b2* expression in the fetal hippocampus was also unchanged following stress; however, *Hsd11b1* and *Hsd11b2* expression is low in the fetal hippocampus in late pregnancy (Moisan *et al.* 1992b, Diaz *et al.* 1998, Wyrwoll *et al.* 2015); thus, local control may be less important at this developmental stage.

Interestingly, greater hepatic corticosterone concentrations were observed in the stressed fetuses, irrespective of sex. This may result from uptake from the maternal or fetal circulation since the liver is perfused by both the umbilical vein (maternal source) and the hepatic portal vein (fetal source) (Murphy *et al.* 2006). It is also possible that corticosterone is generated locally as prenatal stress increases fetal liver *Hsd11b1* expression in mice where it is linked to metabolic dysregulation (Maeyama *et al.* 2015). Previous studies report that maternal stress and increased corticosterone levels affect hepatic gluconeogenic capacity in rat fetuses, potentially leading to the programming of metabolic diseases (Franko *et al.* 2017). Indeed, we previously found alterations in the hepatic expression of genes regulating glucose homeostasis and lipid metabolism in adult prenatally stressed offspring (Brunton *et al.* 2013), suggesting that liver function is particularly sensitive to changes in maternal stress.

In summary, this study demonstrates that repeated maternal social stress stimulates corticosterone secretion in late pregnant rats, though the primary driver of the HPA axis is *Avp*, rather than *Crh*. Despite the increase in circulating corticosterone in the maternal circulation, only a modest increase was detected in the fetal circulation of female, but not male fetuses, with no changes in fetal brain levels of corticosterone in either sex. In contrast to previous reports, repeated maternal social stress did not down-regulate placental *Hsd11b2* or HSD11B2 (Mairesse *et al.* 2007, Jensen Peña *et al.* 2012), suggesting the placental barrier understood to safeguard fetuses from excess maternal glucocorticoid exposure was intact. Maternal stress also exerted a sex-specific increase in the expression of the NR3C1 co-chaperone, *Fkbp51*, in the placenta of female, but not male fetuses, indicating sex differences in responses to stress are evident in prenatal life and further suggesting that mechanisms underpinning fetal programming are likely sex-dependent, with the placenta playing a significant role. In conclusion, our results indicate that any role for maternal glucocorticoids in fetal programming the offspring’s brain and behaviour is likely mediated through indirect actions, perhaps through altering placental gene expression and function in a sex-specific manner, rather than exerting direct effects via crossing the placenta and accessing the fetal brain.

## Supplementary Material

Supplementary Material

## Conflict of interest

The authors have no conflicts of interest to declare.

## Funding

This work was supported by Biotechnology and Biological Sciences Research Council http://dx.doi.org/10.13039/501100000268 (BBSRC) Institute Strategic funding (BB/J004332/1) and the British Society for Neuroendocrinology http://dx.doi.org/10.13039/100014669. YS was supported by a University of Edinburgh Principal's Career Development Scholarship.
