# Supplementary material for: Maternal glucocorticoids do not directly mediate the effects of maternal social stress on the fetus
Source: J Endocrinol. 2022 Oct 18;255(3):143–58. doi: 10.1530/JOE-22-0226 (PMC9716396; doi:10.1530/JOE-22-0226)
Supplement: Supplementary Material [file supplementary_material.pdf]

SUPPLEMENTARY INFORMATION

Maternal glucocorticoids do not directly mediate the effects of maternal social stress on the fetus

Ying Sze[1,2], Joana Fernandes[2], Zofia M. Kołodziejczyk[1] & Paula J. Brunton[1,2,3*]

[1]*Centre for Discovery Brain Sciences; and* [2]*The Roslin Institute, University of Edinburgh, Edinburgh, UK;* [3]*Zhejiang University-University of Edinburgh Institute, International Campus, Haining, Zhejiang, P.R. China.*

SUPPLEMENTARY METHODS

Liquid chromatography (LC) tandem mass spectrometry (MS)

Corticosterone and 11-DHC (#Q1550-000 and #Q3690-000; Steraloids Inc., RI, USA) were first dissolved in methanol, then combined into a 25 ng/ml solution in 0.2% (w/v) bovine serum albumin (BSA). The 25 ng/ml standard solution was then serially diluted 2.5-fold in 0.2% BSA, generating six additional calibration solutions of 10 ng/ml, 4 ng/ml, 1.6 ng/ml, 640 pg/ml, 256 pg/ml and 102.4 pg/ml. 100 µL of each standard calibrant solution was used for processing. The deuterated internal standard corticosterone-d4 (#802905; Sigma, St Louis, MO, USA) was diluted into a working solution of 25 ng/ml in 50% methanol and 20 µL was used for each sample.

Frozen tissue samples (one hemisphere of a fetal brain, 1/8th of a placenta or 50mg of fetal liver) were weighed and plasma was diluted 1:100 before sample processing. Tissue samples were homogenised in 500 µL of methanol/1% formic acid (FA), while 400 µL of methanol/1% FA was added to 100 µL of diluted plasma or standard calibrants. 20 µL of 25 ng/ml corticosterone-d4 was added to all samples and calibrants as an internal standard. Homogenates were sonicated, incubated on dry ice, and then centrifuged. The supernatant was collected and the pellet was resuspended with another 500 µL of methanol/1% FA for a second round of homogenisation, sonication and centrifugation. Combined supernatants were then diluted to a final concentration of 30% methanol, and loaded on solid phase extraction columns (Supelco Discovery DSC-18 SPE Cartridge #52602-U, Sigma, UK), which had been pre-activated with 1 mL of methanol and 1 mL of 30% methanol. After two 1 mL washes with 40% methanol, steroids were eluted with 1 mL of 85% methanol. Eluates were dried in a vacuum overnight before derivatisation, where 400 µL of 1 mg/mL of Girard's T

reagent (Sigma #89397; dissolved in methanol/0.2% FA) was added. The solution was incubated for 30 min at 37°C, followed by the addition of 50 µL of 5% ammonium hydroxide in methanol to stop the reaction. Samples were dried, then reconstituted in 50 µL of 50% methanol for LC-MS analysis.

LC-MS analysis was performed using an Ultimate 3000 Dionex HPLC system (Thermo Fisher, Waltham, MA, USA) with a refrigerated autosampler (8°C), coupled to an AmaZon ETD ion trap mass spectrometer (Bruker Daltonics, Bremen, Germany). The ACE UltraCore 2.5µm Super C18 column (#CORE-25A-7502U; 75 mm by 2.1 mm inner diameter; Advance Chromatography Technologies, Aberdeen, UK) was used for reverse phase HPLC, with 50mM ammonium formate (pH 3) and methanol/0.1% FA as mobile phase A and B respectively. Transitions (with positive electrospray ionisation and collision-induced dissociation) were monitored for corticosterone (460.2 → 401.1), 11-DHC (458.2 → 399.2) and corticosterone-d5 (464.3 → 405.1). Data were acquired using the Hystar software and the peak area under curve (AUC) was extracted and automatically integrated using the QuantAnalysis software (both Bruker Daltonics). The ratio of the AUC (corticosterone or 11-DHC): AUC (corticosterone-d4) was used to construct calibration curves, with linear regression and a weighting of 1/x. Concentrations of samples were extrapolated, corrected for the dilution factor and converted to ng/ml (for plasma), or normalised to the wet weight of the tissues (ng/g for brain, liver and placenta).

***In situ* hybridisation**

15 µm cryostat sections were thaw-mounted onto Polysine® coated slides and stored at −80°C until processing. Maternal and fetal brains were cut coronally, whilst placenta was cut transversally to visualise both junctional and labyrinth zones. Sections were fixed with 4% paraformaldehyde, acetylated and dehydrated on the day of the hybridisation.

For oligonucleotide probes, cDNA probes complementary to arginine vasopressin (*Avp*) and pro-opiomelanocortin (*Pomc*) were purchased from Eurofins Genomics (Ebersberg, Germany) and 3’-end labelled with [^{35}S]-dATP (PerkinElmer, #NEG034H250UC) using terminal deoxynucleotidyl transferase and purified using the QIAquick nucleotide removal kit (Qiagen). Radiolabelled probes were then diluted in hybridisation buffer, applied to tissue sections and incubated overnight in humidified chambers at 37°C. After hybridisation, sections underwent post-hybridisation washes (Suppl. Table 1A), dehydration, and then were dipped in liquid autoradiographic emulsion and exposed in the dark at 4°C (Suppl. Table 1A).

ISH for corticotropin-releasing hormone (*Crh*), glucocorticoid receptor (*Nr3c1*), mineralocorticoid receptor (*Nr3c2*), 11β-hydroxysteroid dehydrogenase type 1 (*Hsd11b1*) and type 2 (*Hsd11b2*), FK506-Binding Protein 51 (*Fkbp51*) and FK506-Binding Protein 52 (*Fkbp52*) was carried out using riboprobes. Plasmids containing the cDNA of the genes of interest were linearised with specific restriction enzymes (Suppl. Table 1B) and reverse transcribed using specific polymerases (Promega Riboprobe Systems; Suppl. Table 1B) to generate [^{35}S]-UTP (Perkin-Elmer #NEG039H250UC) labelled probes. In each case, antisense riboprobes were used to detect mRNA expression, while sense riboprobes were also generated and used as negative controls (Figure S1 for *Hsd11b2*). Radiolabelled riboprobes were purified using Illustra NICK Columns (GE Healthcare Life Sciences, UK), diluted in hybridisation buffer and applied to tissue sections, which had undergone a 2h pre-hybridisation step. Hybridisation occurred overnight at 55°C in humidified chambers. After hybridisation, the sections were washed briefly in 2x saline sodium citrate (SSC) buffer, before RNase A treatment for 60 min, and then post-hybridisation washes in heated SSC buffer of decreasing concentrations (Suppl. Table 1B). Sections were then dehydrated and dipped in liquid autoradiographic emulsion and exposed in the dark at 4°C (Suppl. Table 1B). In each case, following the appropriate exposure time, emulsion-dipped sections were developed, fixed, then counterstained with haematoxylin and eosin and cover-slipped.

Hybridised sections were visualised on the Nikon Ni2, Leica DMR, or the Hamamatsu Nanozoomer microscopes and TIFF images were acquired on the Zen 2 blue (Carl-Zeiss), LASX (Leica Microsystems) or NDP.view2 software (Hamamatsu), respectively. Images were converted to 16-bit and analysed using Fiji software. The number of positive cells (defined as cells expressing silver grains at a density ≥5x higher than that of the background), grain density or average pixel intensity was measured, depending on the probe and region of interest. For grain density and pixel intensity, the threshold was manually adjusted on Fiji to select for the area containing grains. A second measurement which included the underlying cells and tissues (i.e. total area sampled) was made in order to obtain density (mm^2/mm^2). For gene expression analysis, 3-4 sections/rat were analysed bilaterally across the entire region of interest for the maternal brain and 6-8 sections/rat were analysed bilaterally for the fetal brain. Gene expression in the anterior pituitary gland was analysed in 3 sections/rat using 15-20 sampling frames across the entire region. In the placenta, gene expression was analysed in 2-3 sections/rat using 20-30 sampling frames across the entire region of interest.

TABLE 1A

Target mRNA	Sequence (5' to 3')	Post-hybridisation washes	Exposure time
Avp	GACCCGGGGCTTGGCAGAATCCACGGACTCTTGTGT	1X SSC: 4x15min (58°C)<br>2x30min (RT)	7d
Pomc	CATGAAGCCCGCCTAGCGCTTGTCCTTGGGCGGGTTGCCCCAGCG	1X SSC: 4x15min (63°C)<br>2x30min at RT	35d

TABLE 1B

<table>
<tr><th>Target mRNA</th><th>Insert size (bp)</th><th colspan="2">Restriction enzymes</th><th colspan="2">RNA polymerase</th><th>Post-hybridisation washes</th><th>Exposure time</th><th>Ref.</th></tr>
<tr><td>Crh</td><td>518</td><td>AS: XbaI</td><td>S: HindIII</td><td>AS: T3</td><td>S: T7</td><td rowspan="5">2X SSC: 30min (RT)
0.1X SSC: 3x1h (60°C)</td><td>25d</td><td>1</td></tr>
<tr><td>Nr3c1</td><td>620</td><td>AS: AvaI</td><td>S: EcoRI</td><td>AS: T7</td><td>S: SP6</td><td>28d</td><td>2</td></tr>
<tr><td>Nr3c2</td><td>513</td><td>AS: HindIII</td><td>S:EcoRI</td><td>AS: SP6</td><td>S: T7</td><td>28d</td><td>2</td></tr>
<tr><td>Hsd11b1</td><td>616</td><td>AS: NotI</td><td>S: KpnI</td><td>AS: T7</td><td>S: SP6</td><td>35d</td><td>3</td></tr>
<tr><td>Hsd11b2</td><td>750</td><td>AS: SphI</td><td>S: SalI</td><td>AS: SP6</td><td>S: T7</td><td>28d</td><td>4</td></tr>
<tr><td>Fkbp51</td><td>491</td><td>AS: BamH1</td><td>S: Xho</td><td>AS: T3</td><td>S: T7</td><td rowspan="2">2X SSC: 30min (RT)
0.1X SSC: 2x30min (RT)
0.1X SSC: 1h (65°C)</td><td>28d</td><td>5</td></tr>
<tr><td>Fkbp52</td><td>157</td><td>AS: BamH1</td><td>S: Xho</td><td>AS: T3</td><td>S: T7</td><td>16d</td><td>6</td></tr>
</table>

Supplementary Table 1: Probe-specific details for *in situ* hybridization (ISH) using (A) oligoprobes and (B) riboprobes. Abbreviations: AS, antisense; bp, base pairs; RT, room temperature; S, sense; SSC, saline sodium citrate buffer.

Figure S1

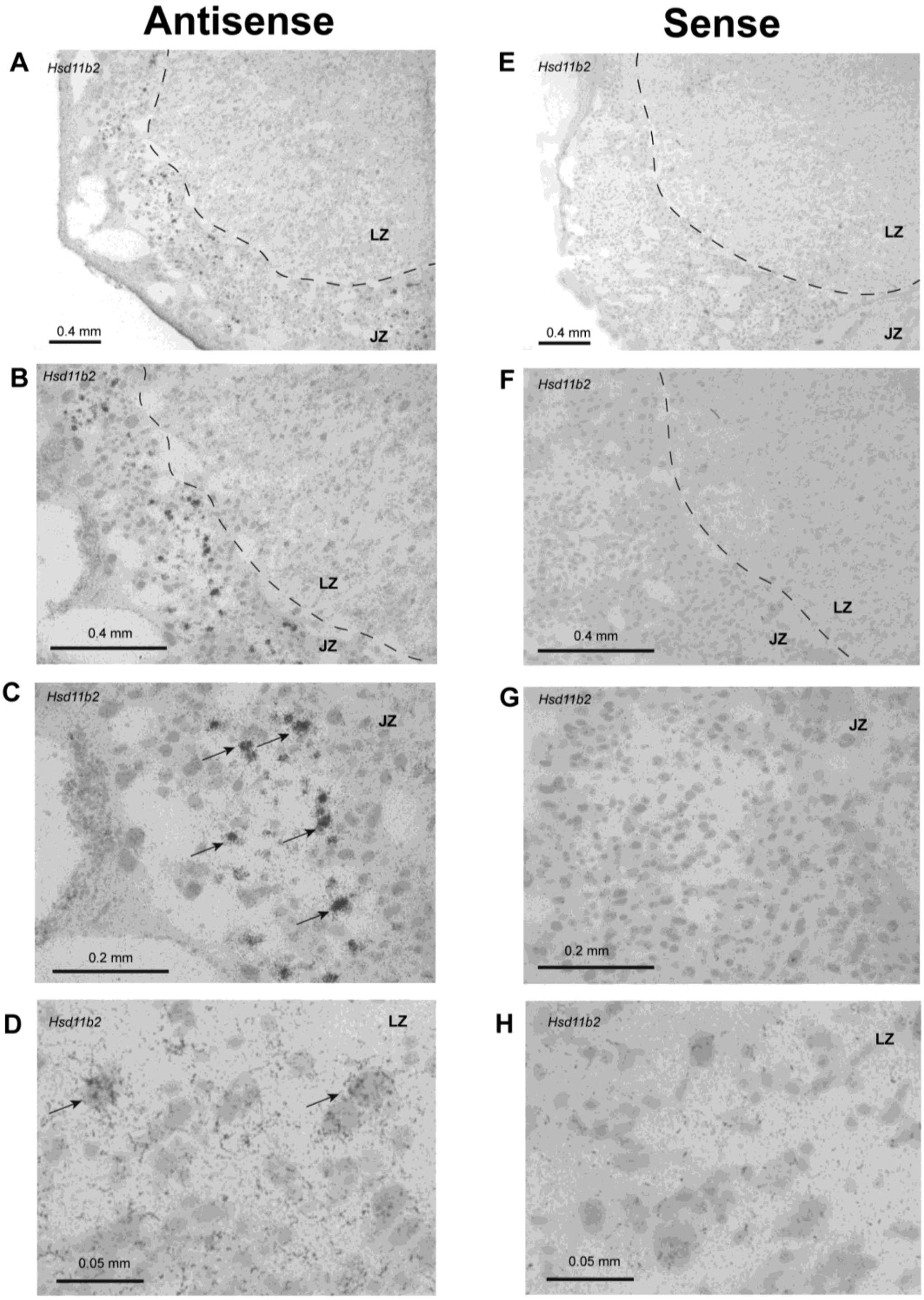

Figure S1: Representative images of *in situ* hybridization with antisense (A-D) and sense (E-H) probes for placental *Hsd11b2*. Clusters of silver grains were observed overlaying cells (black arrows) in A-D. No signal above background was detected in tissue hybridised with sense probes (E-H). The dotted line demarcates the junctional zone (JZ) and labyrinth zone (LZ) in A, E (5x magnification) and B, F (10x magnification). C and G: Image taken at 20x magnification in the JZ. D and H: Image taken at 20x magnification in the LZ and further digitally zoomed to visualise individual silver grains over cells.

Western blotting for placental 11β-HSD2

Sample homogenisation and protein quantification: 1/8th of a placenta, comprising both junctional and labyrinth zones, was excised and homogenised on ice in RIPA lysis buffer with HALT protease inhibitor (both ThermoFisher) and centrifuged at 4°C for 20 min at 10000 g. Supernatants were removed and protein concentration determined using a Micro BCA Protein Assay Kit (ThermoFisher #23235), according to manufacturer's instructors. Placental supernatants were further diluted 1:40 with PBS and analysed alongside standard calibrants with concentrations of 125 – 2000 µg/ml (serially diluted from 2 mg/ml BSA protein standards; Sigma). After incubation with the BCA working reagent at room temperature for 2h, optical density was read at 570 nm and the protein concentration of each sample was determined via a linear standard curve.

SDS-PAGE and semi-dry transfer: 50 µg of protein per placental sample was incubated at 70ºC for 10 min with NuPAGE reducing agent (10x) and loading buffer (4x; LDS sample buffer containing loading dye). Samples, together with a MagicMark XP protein ladder, were then loaded on pre-cast NuPAGE 4-12% Bis-Tris protein gels. Electrophoresis was carried out at 85V for 15 min and then at 185V, with 1X NuPAGE MOPS SDS as running buffer on the XCell SureLock Mini Cell platform. Semi-dry transfer was then performed on a Novex Semi-Dry Blotter (ThermoFisher Scientific) with 0.1M Tris, 0.2M glycine, 5% (v/v) methanol as transfer buffer, onto Immobilon-FL PVDF Membranes (Merck Millipore). Transfer was carried out at 280 mA for 1h, and membranes were air-dried after transfer to permanently bind proteins to the membrane.

Immunoprobing and detection: Dried PVDF membranes were reactivated in methanol for 2 min, rinsed with ddH_2O, and then with PBS for 2 min. Membranes were then blocked in Odyssey blocking buffer (Li-Cor Bioscience, Cambridge, UK) for 1h and incubated at 4°C overnight with primary antibody targeting the 11β-HSD2 protein (Abcam, #ab80317; 1:250 diluted in blocking buffer with 0.1% (v/v) Tween-20). Membranes were then washed in PBS (10 min x 5) and incubated with a fluorescent secondary antibody (Goat anti-rabbit IgG IRDye 680RD, Li-Cor, 1:5000) for 1h at RT. Following another set of 5 x 10 min washes with PBS, blots were visualised on the Li-Cor Odyssey Infrared Imaging System. To probe for the internal loading control β-actin, the blots were then stripped for 20 min using a mild stripping buffer (15 g/L glycine, 1 g/L SDS, 1% v/v Tween 20, protocol from Abcam), then washed twice with PBS. Membranes were incubated with primary antibody targeting β-actin (Sigma, #A5411; 1:50000), for 1h at RT, washed, and then incubated with fluorescent secondary antibody (Donkey anti-mouse IgG IRDye 680 RD, Li-Cor, 1:10000) for 1h at RT, and visualised as before.

Data analysis: Densitometric analyses were carried out using ImageJ (NIH, Washington, DC), using commands from the “Analyze→Gels” submenu, where both the size and the grey density of each band was taken into account. The grey value obtained for 11β-HSD2 was then normalised to the grey value obtained for β-actin. Two technical duplicates were run for every sample (i.e. two independent Western blot runs), and a mean value was calculated.

REFERENCES

1. Harris, H.J., et al., Intracellular regeneration of glucocorticoids by 11beta-hydroxysteroid dehydrogenase (11beta-HSD)-1 plays a key role in regulation of the hypothalamic-pituitary-adrenal axis: analysis of 11beta-HSD-1-deficient mice. *Endocrinology*, 2001. **142**: 114-20.
2. Seckl, J.R., K.L. Dickson, and G. Fink, Central 5,7-dihydroxytryptamine lesions decrease hippocampal glucocorticoid and mineralocorticoid receptor messenger ribonucleic Acid expression. *J Neuroendocrinol*, 1990. **2**: 911-6.
3. Agarwal, A.K., et al., Cloning and expression of rat cDNA encoding corticosteroid 11 beta-dehydrogenase. *J Biol Chem*, 1989. **264:** 18939-43.
4. Leckie, C., et al., LLC-PK1 cells model 11 beta-hydroxysteroid dehydrogenase type 2 regulation of glucocorticoid access to renal mineralocorticoid receptors. *Endocrinology*, 1995. **136**: 5561-9.
5. Scharf, S.H., et al., Expression and Regulation of the Fkbp5 Gene in the Adult Mouse Brain. *PLos One*, 2011. **6**(2).
6. Hartmann, J., et al., The involvement of FK506-binding protein 51 (FKBP5) in the behavioral and neuroendocrine effects of chronic social defeat stress. *Neuropharmacology*, 2012. **62**: 332-9.
